# Evidence of high-elevation amplification versus Arctic amplification

**DOI:** 10.1038/srep19219

**Published:** 2016-01-12

**Authors:** Qixiang Wang, Xiaohui Fan, Mengben Wang

**Affiliations:** 1 Institute of Loess Plateau, Shanxi University, Taiyuan 030006, China

## Abstract

Elevation-dependent warming in high-elevation regions and Arctic amplification are of tremendous interest to many scientists who are engaged in studies in climate change. Here, using annual mean temperatures from 2781 global stations for the 1961–2010 period, we find that the warming for the world’s high-elevation stations (>500 m above sea level) is clearly stronger than their low-elevation counterparts; and the high-elevation amplification consists of not only an altitudinal amplification but also a latitudinal amplification. The warming for the high-elevation stations is linearly proportional to the temperature lapse rates along altitudinal and latitudinal gradients, as a result of the functional shape of Stefan-Boltzmann law in both vertical and latitudinal directions. In contrast, neither altitudinal amplification nor latitudinal amplification is found within the Arctic region despite its greater warming than lower latitudes. Further analysis shows that the Arctic amplification is an integrated part of the latitudinal amplification trend for the low-elevation stations (≤500 m above sea level) across the entire low- to high-latitude Northern Hemisphere, also a result of the mathematical shape of Stefan-Boltzmann law but only in latitudinal direction.

It is well-known that the warming for the Arctic region is greater than at lower latitudes^1^,^2^,^3^,^4^,^5^,^6^,^7^. However, despite the fact that multiple lines of evidence suggest that high-elevation areas are warming faster than their lower elevation counterparts across the globe, it is not yet definitive how much faster^8^,^9^,^10^,^11^,^12^. It is also unclear how warming in high-elevation regions differs from in the Arctic region. Recently, Wang *et al.*^13^ found that the warming for the four high-elevation regions is on average 1.26 times faster than their lower elevation counterparts based on a paired region comparison method. Meanwhile, these authors uncovered a significant altitudinal amplification trend for each of the eight high-elevation regions tested with a new methodology they developed. Here, using annual mean temperatures from 2781 global stations (Fig. 1) for the 1961–2010 period, we quantify how much faster the warming is for the world’s high-elevation stations (>500 m a.s.l) as a whole in comparison with their low –elevation counterparts (≤500 m a.s.l) based on some standard methods. Similar analysis is also performed for the Arctic stations (North of 60 °N) as a whole in comparison with their low-latitude counterparts in order to test how high-elevation areas differ from the Arctic region.

We begin with comparing the temperature trends between the high-elevation stations (Ele-high) and their low-elevation counterparts (Ele-low) and between the low-elevation stations in the Arctic region (Lat-high) and their lower latitude counterparts (Lat-low) using a method similar to the paired region comparison method^13^. We examine the elevation-dependent warming along altitudinal gradient and the latitude-dependent warming along latitudinal gradient using a method similar to the elevation band method^14^,^15^,^16^ but with quantification of the amplification trend. We focus on the examination of the relationship of temperature trends with altitude and latitude for the Ele-high compared with the Ele-low, and for the Lat-high relative to all the low-elevation stations across the entire low- to high-latitude Northern hemisphere (NH-Ele-low) using the stepwise regression method. At the same time, we evaluate the sensitivity of altitudinal amplification and latitudinal amplification trends to changes of altitude and number of available stations by re-sampling the data of the Ele-high, as well as the sensitivity of latitudinal amplification trend to changes of latitude and number of available stations by re-sampling the data of the NH-Ele-low. See Supplementary Tables S1 and S2 for details on the descriptive statistics and the proportions of positive and negative trends for each of the six groups of stations tested.

## Results

**Figure 2:**
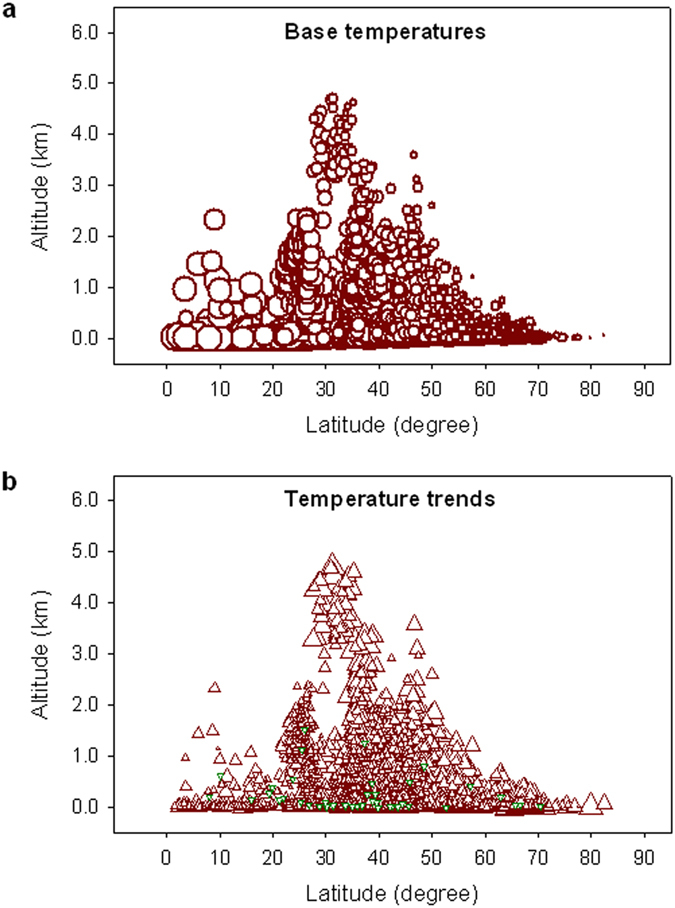
Change patterns of base temperatures and temperature trends with altitude and latitude for the 2781 available stations around the globe. (**a**) Dark red circles, from largest to smallest, represent the base annual mean temperatures at individual stations from 30 °C to −20 °C in intervals of 5 °C. (**b**) Dark red upward triangles, from smallest to largest, represent 50-year warming trends from 0 °C to about 3.5 °C 50-yr^−1^ in intervals of 0.5 °C, while dark green downward triangles stand for cooling trends down to about −1.0 °C 50-yr^−1^over the period 1961–2010.

**Figure 3:**
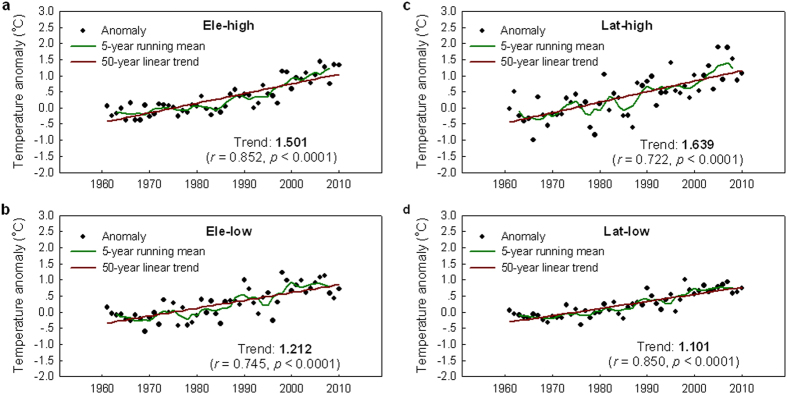
High-elevation amplification and Arctic amplification. Linear trends of annual mean temperatures (1961–2010) over the period 1961–2010 for (**a**) the high-elevation stations (**Ele-high**) compared with (**b**) their lower elevation counterparts (**Ele-low**) located in the same latitudes (3.40 °N/S−63.25 °N/S), and for (**c**) the low-elevation stations in the Arctic region (north of 60 °N, **Lat-high**) compared with (**d**) their lower latitude counterparts (**Lat-low**) at the same altitudes (0−500 m a.s.l). The trend is expressed in °C 50-yr^−1^. Significant trend, at the 95% confidence level, is set in bold (see Methods for details on calculations of the trends).

Because all the stations are distributed in the shape of a pyramid along the entire altitudinal gradient, and the changes of base temperatures (the 1961–1990 average, ***T***_**b**_) and temperature trends (***T***_**t**_) appear associated with both altitude and latitude (Fig. 2), the comparison of temperature trends is performed for the paired high- and lower-elevation stations located in the same latitudes, while for the paired high- and lower-latitude stations located in the same altitudes. Results show that the rate of warming for the Ele-high is about 1.24 times faster than the Ele-low over the period 1961–2010 (Fig. 3a,b); and the rate of warming for the Lat-high is about 1.49 times faster than the Lat-low over the same period (Fig. 3c,d). The 50-year warming trend for the Lat-high is comparable to that (1.80 °C 50-yr^−1^) for 1959–2008 in the Arctic region^3^.

**Figure 4:**
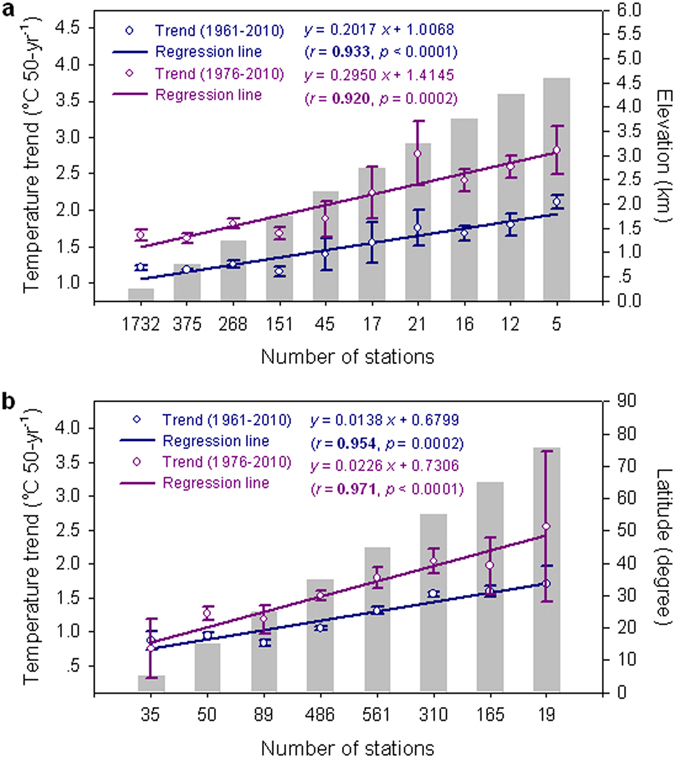
Elevation-dependent warming and latitude-dependent warming. (**a**) Elevation-dependent warming for the 910 high-elevation stations and their 1732 low-elevation counterparts located in the same latitudes (3.40 °N/S−63.25 °N/S). Bars represent elevation, and trend magnitude is plotted on the y axis according to the 10 elevation ranks of 2642 stations. (**b**) Latitude-dependent warming for the 184 low-elevation stations in the Arctic region (north of 60 °N) and their 1531 lower latitude counterparts (0 °N−60 °N) at the same altitudes (0−500 m a.s.l). Bars represent latitude, and trend magnitude is plotted on the y axis according to the 8 latitude ranks of 1715 stations. Error bars denote one standard error around the mean. Pearson correlation coefficient (*r*) between trend magnitude and elevation (latitude) is shown with two-tailed *p* value. Significant coefficient, at the 95% confidence level, is set in bold.

Moreover, systematic increases in warming rate with elevation are found for the Ele-high relative to the Ele-low, especially for the period 1976–2010 (Fig. 4a). A significant altitudinal amplification trend is detected along the entire altitudinal gradient for both 1961–2010 and 1976–2010, and the rate of altitudinal amplification over the last 35 years is 1.46 times that for the last 50 years (Fig. 4a). An analogous method has been used to present the relationship between temperature trends and elevation on and around the Tibetan Plateau in previous studies^14^,^15^,^16^. However, no statistical significance was shown^14^,^16^ or no significance test was performed^15^. Therefore, while the warming rates appear, to various extents, to be amplified with elevation, it is uncertain whether the relationship is statistically significant. For the NH-Ele-low, the increases in warming rate with latitude are evident across the entire low- to high-latitude Northern Hemisphere over the period 1961–2010 (Fig. 4b). A significant latitudinal amplification trend is found along the entire latitudinal gradient for both entire (1961–2010) and recent (1976–2010) periods, and the rate of latitudinal amplification over the last 35 years is 1.64 times that for the last 50 years (Fig. 4b).

Notably, stepwise regression analysis reveals a significant positive relationship between *T*_t_ and altitude and latitude for the Ele-high, while a significant positive relationship between *T*_t_ and latitude for the Ele-low, for which the effect of altitude is not significant (Table 1). It can be seen from the linear models for these two groups of stations that the warming for the Ele-high is not only closely associated with altitude but also latitude, while the warming for the Ele-low is only related to latitude. This indicates that there is not only a significant altitudinal amplification trend of 0.2297 °C km^−1^ 50-yr^−1^ but also a significant latitudinal amplification trend of 0.2510×10^−3^ °C km^−1^ 50-yr^−1^ for the Ele-high over the past 50 years, while there is only a significant latitudinal amplification trend of 0.1925×10^−3^ °C km^−1^ 50-yr^−1^ for the Ele-low in the same period. Moreover, the faster rate of latitudinal amplification for the former than the latter indicates that the latitudinal amplification trend for the former is an enhanced result of the base latitudinal amplification trend for the latter due to increase of altitude. Therefore the warming amplification for the Ele-high is essentially a combination of an altitudinal amplification trend and an enhanced base latitudinal amplification trend.

**Figure 5:**
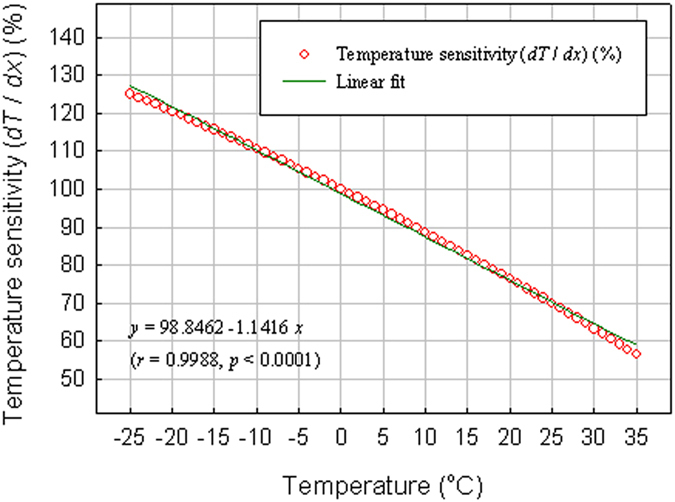
Temperature sensitivity as a function of temperature. Red circle denotes the temperature sensitivity (*dT/dx*), expressed as the percentage of that at 0 °C. Dark green line stands for the linear regression line. Based on the Stefan-Boltzmann equation ∑*F*_*i*_ = σ*T*^4^, *dT/dx* = (1/4σ*T*^3^) (∑*dF*_*i*_/*dx*), where *F*_*i*_ is the *i*th energy flux (*i* = 1 to *n*), *T* is temperature, σ is Stefan-Boltzmann constant, and *x* is a given energy flux in a general sense. Suppose a change in *x* causes changes in energy fluxes exactly in the same manner in warmer and colder climates, and the energy flux changes (∑*dF*_*i*_/*dx*) are the same for these climates, the temperature sensitivity (*dT/dx*) at lower temperature is larger than at higher temperature, as a result of *T*^3^ in the denominator^17^. For instance, the temperature sensitivity at 0 °C is 25% smaller than at −25 °C but 30% larger than at 25 °C.

Furthermore, from the linear models established for the Ele-high, that is, *T*_t_ = *b*_0_ *T*_b_ + *c*_1_, *T*_b_ = *b*_1_ *x*_1_ + *b*_2_ *x*_2_ + *c*_2_ (equations (1.2) and (1.3) in Table 1), it can be derived that *T*_t_ = *b*_0_*b*_1_ *x*_1_ + *b*_0_*b*_2_ *x*_2_ + *c*_4_. This new model is in fact another format of the model *T*_t_ = *b*_3_ *x*_1_ + *b*_4_ *x*_2_ + *c*_3_ (equation (1.1) in Table 1). This implies that the warming for the Ele-high is linearly proportional to the temperature lapse rates along altitudinal and latitudinal gradients. As illustrated in Fig. 5, the effect of energy balance variation on the surface temperature can be amplified with decreasing temperature in the environment, as a result of the functional shape of Stefan-Boltzmann law^17^. This suggests that the high-elevation amplification could be a consequence of the mathematical shape of Stefan-Boltzmann law in both vertical and latitudinal directions.

As for the Ele-high, the relationship of *T*_t_ with altitude and latitude was also analyzed for the Arctic stations. However, no significant relationship between *T*_t_ and altitude and/or latitude is found for these stations (Table 1), suggesting there is no altitudinal amplification or latitudinal amplification within the Arctic region. Despite this fact, however, further analysis shows a significant positive relationship between *T*_t_ and latitude for the NH-Ele-low (Table 1), indicating a significant latitudinal amplification trend across the low- to high-latitude Northern Hemisphere. This suggests that the Arctic amplification is an integrated part of the latitudinal amplification trend for the NH-Ele-low. Similarly, from the linear models established for the NH-Ele-low, that is, *T*_t_ = *b*_0_ *T*_b_ + *c*_1_, *T*_b_ = *b*_2_ *x*_2_ + *c*_2_ (equations (4.2) and (4.3) in Table 1), it can be derived that *T*_t_ = *b*_0_*b*_2_*x*_2_ + *c*_4_. This new model is in fact another format of the model *T*_t_ = *b*_4_ *x*_2_ + *c*_3_ (equation (4.1) in Table 1). This implies that the warming for the NH-Ele-low is linearly proportional to the temperature lapse rate along the latitudinal gradient. This result suggests that the latitudinal amplification trend on the hemispheric scale, as well as the Arctic amplification, is probably a consequence of the functional shape of Stefan-Boltzmann law, but only in the latitudinal direction.

Turning to the examination of the sensitivity of altitudinal amplification and latitudinal amplification trends to altitude and available stations, as shown in Supplementary Table S3, the altitudinal amplification and latitudinal amplification trends are only detected for the first 17 and 16 altitude extents sampled, respectively. Afterwards, neither altitudinal amplification trend nor latitudinal amplification trend can be detected further despite even larger average temperature trends. At the same time, as shown in Supplementary Fig. S1, it can be seen that the rates of the last three altitudinal amplification trends and the last two latitudinal amplification trends detected drops dramatically compared to that just before despite increasing warming trends. This suggests that, with the increase of average altitude, and the decrease of altitude (latitude) extent, there should be a critical altitude (latitude) extent for the detection of altitudinal (latitudinal) amplification trend; and an even larger critical altitude (latitude) extent for an accurate estimation of altitudinal (latitudinal) amplification trend.

Why is there such a threshold effect? As shown in Supplementary Fig. S2, with the omission of lower elevation bands, the average altitude increases, whereas the number of available stations decreases sharply. Predominately controlled by this decrease, the warming signal quantity (WSQ) diminishes dramatically at the same time. Consequently, the altitudinal and latitudinal warming signal quantity (WSQ_alt_ and WSQ_lat_, respectively) decreases sharply. When the WSQ_alt_ (the number of stations) falls below the critical value of about 100 °C 50-yr^−1^(about 120), the rate of altitudinal amplification trend becomes abnormally small until no altitudinal amplification trend can be detected anymore. When the WSQ_lat_ (the number of stations) falls below the critical value of about 75 °C 50-yr^−1^(about 95), no latitudinal amplification trend can be detected further. This indicates that the threshold effect is essentially a reflection of the threshold effect of warming signal quantity (the number of stations).

For the NH-Ele-low, the evaluation of the sensitivity of latitudinal amplification trends to latitude and available stations show a similar result (Supplementary Table S4 and Supplementary Fig. S3). The critical warming signal quantity (the critical number of stations) for the detection of latitudinal amplification trend is about 915 °C 50-yr^−1^ (580). Therefore, no latitudinal amplification trend can be detected within the Arctic region.

For the detection of latitudinal amplification trend, the value of the critical warming signal quantity obtained from sampling the NH-Ele-low is obviously larger compared with that revealed from sampling the Ele-high. This is due to the substantial difference in the areas across which the sampled stations are distributed. The land area above 2.2 km a.s.l is about 6.3 million km^2^ according to the global pattern of land area outside Antarctica per altitude in 100 m steps a.s.l.^18^, while the land area in the north of 47.5 °N is over 35 million km^2^. Whereas a general comparison can only be made using signal intensity (per unit area signal quantity) rather than signal quantity. Nevertheless, from these two sampling experiments, it can be derived that even if the warming is very strong for a high-elevation region (or a high-latitude region), a certain number of stations are still required for the detection of altitudinal amplification and latitudinal amplification trends (or latitudinal amplification trend) and for an accurate estimation of altitudinal amplification and latitudinal amplification trends (or latitudinal amplification trend), the number of stations required is even larger.

## Discussion

Pepin and Lundquist^19^ have analyzed the relationship between *T*_t_ and altitude for the world’s 1084 high-elevation stations (>500 m above sea level) as a whole, but found no strong correlation between *T*_t_ and altitude. As Pepin and Seidel^20^ described in an earlier study, the main trend analyses for the entire study period 1948–2002 were restricted to the period 1948−1998, and the median trend for the 1084 high elevation stations is 0.13 °C per decade over the period 1948–1998, with 444 (41%) of the stations showing significant positive trends. In the current study, however, the median trend for the 910 high-elevation stations is 0.25 °C per decade over the period 1961–2010, with 786 (86.4%) of the stations showing significant positive trends. A significant relationship between altitude and *T*_t_ is uncovered for these stations in the last 50 years (*r* = 0.200, *p* < 0.0001). Meantime, analysis for the period 1961–1998 reveals no strong relationship between altitude and *T*_t_ for the 910 high-elevation stations (*r* = 0.037, *p* = 0.263), even though the median trend for them is 0.17 °C per decade in this period, with 421 (46.3%) of the stations showing significant positive trends. Therefore, the failure in quantifying altitudinal amplification trend in the previous study^19^ is primarily due to the weaker warming over 1948–1998 than 1961–2010, or because the decade of 2000’s, when warming was stronger than for any the previous decades of the instrumental record^21^, was not covered.

Further, it is worth noting that when the relationship of *T*_t_ with altitude and latitude is tested for the Ele-high, a significant positive relationship can be detected for every time period longer than 30 years starting from 1961 during the period 1961–2010. For instance, a significant positive relationship is detected for 1961–1995 (*R* = 0.456, *p* < 0.0001) and 1961–2005 (*R* = 0.469, *p* < 0.0001), with the altitudinal amplification trend of 0.0307 °C km^−1^ per decade and 0.0330 °C km^−1^ per decade, respectively. The rates of altitudinal amplification trends are 0.33 and 0.28 times smaller than that (0.0459 °C km^−1^ per decade) for 1961–2010. This is because both the effects (signals) of altitude and latitude and the interacting effect (signal) of altitude and latitude are taken into consideration when *T*_t_ is regressed against these two variables, whereas only the effect (signal) of altitude is taken into consideration when *T*_t_ is regressed against altitude alone.

Climate in mountainous regions is proposed to be controlled by four principal factors, i.e., altitude, latitude, continentality, and topography^22^. Altitude and latitude are proved to be major factors in determining the geographical pattern of temperature change in the Alps^23^. The current study confirms further that both altitude and latitude are key factors in shaping the global-scale high-elevation amplification, and that latitude is the dominant factor of Arctic amplification. Notably, our results suggest that the Stefan-Boltzmann law could be a key mechanism that induces the amplifications of warming at high-elevations and in the Arctic region.

Several physical processes have been suggested to explain Arctic amplification^4^. It is widely accepted that changes in the surface albedo associated with declining sea ice and snow cover enhance warming in the Arctic^2^,^4^,^5^,^24^,^25^, but other processes may play a part. For example, it has been suggested that changes in cloud cover and atmospheric water vapor content^26^,^27^,^28^, and changes in atmospheric heat transport^1^ may be more important for Arctic amplification than the snow and ice albedo feedbacks. Using an energy balance model, Izumi *et al.*^29^ reveal that surface downward clear-sky longwave radiation (influenced by atmospheric water vapor, moist static energy transport, and CO^2^ concentration) is the most important component driving high-latitude amplification, though surface albedo also plays a significant role in this regard. The distinct latitudinal amplification trend across the low- to high-latitude Northern Hemisphere observed in this study suggests that much of the present Arctic warming is likely a result of the functional shape of Stefan-Boltzman law in latitudinal direction. From the view of response scale, this result is in agreement with the notion that the recent Arctic amplification is mainly originated from the large-scale processes such as enhanced northward heat and moisture transports^1^,^30^ rather than the local-scale surface albedo feedback mechanisms^3^.

Snow-albedo feedback has also been shown to play a significant role in generating the high-elevation amplification^31^,^32^. However, its relative importance remains uncertain compared with other processes such as changes in cloud cover, atmospheric water vapor, and aerosols. For instance, it has been suggested that increases in downward longwave radiation caused by increasing atmospheric water vapor, combined with increases in absorbed solar radiation caused by decreases in snow cover extent, are partly responsible for recent large warming trend over the Tibetan Plateau^33^. In a global study, Ohmura^17^ found that 11 out of 18 regions show a larger warming rate at the summit of mountains during the last 30 to 40 years. He ascribed the high-altitude amplification to two primary processes: the increasing diabatic process in the mid- and high troposphere as a result of the cloud condensation, and the amplifying process in the effect of the energy balance variation on the surface temperature as a result of the functional shape of Stefan-Boltzmann law. In the current study, we find a distinct relationship of the high-elevation warming with the temperature lapse rates along altitudinal and latitudinal gradients. This indicates that much of the high-elevation warming is probably a result of the functional shape of Stefan-Boltzmann law in both vertical and latitudinal directions. However, the question what processes (energy flux terms) are the most important components in the surface energy balance requires further investigation.

## Methods

### Data

The data used in this study consisted of 1,860, 464, 360, 72 and 25 station series (1961–2010) derived from the quality controlled adjusted Global Historical Climatology Network monthly mean temperature dataset (GHCNM version 3.2.0)^34^; the daily mean air temperature data set of the National Meteorological Information Center of China (NMICC), the Daily Temperature and Precipitation Data for 518 Russian Meteorological Stations (RMS)^35^, the Historical Instrumental Climatological Surface Time Series of the Greater Alpine Region (HISTALP)^36^, and MeteoSwiss, respectively. The procedure for establishing the series of annual mean temperature from the raw daily and monthly data was the same as in the previous study^13^. Each of the station series had at least 37 years of records (with twelve months of monthly mean temperature in each year) during the period 1961–2010.

The overall quality of the data from these five sources is fairly good. The monthly data from the GHCNM version 3.2.0 have been quality controlled and adjusted, and the data from the HISTALP and MeteoSwiss have been homogenized. So the annual data series derived from these three sources were used for trend estimation without further homogeneity test. For the annual time series derived from the daily data collected from the NMICC and the RMS, each annual time series was checked for homogeneity using RHtests V3^37^. After removing the stations with inhomogeneous series, 464 and 360 station series from these two sources were used for trend estimation.

The trend for each station was estimated from the anomalies (relative to the 1961–1990 average) using the least squares best-fit method. Of all the stations used for this study, 2348 (84.4%) and 395 (14.2%) show significant positive, and non-significant positive trends, respectively; while 6 (0.2%) and 32 (1.2%) show significant negative, and non-significant negative trends, respectively (see Supplementary Table S2 for details on the trends for each of the six groups of stations used in this study).

### Comparison of temperature trends between high- and low-elevation (latitude) stations

A method based on the principle of paired region comparison^13^ was used for this end. It was performed for the Ele-high and the Ele-low located in the same latitudinal band (3.40°N/S−63.25°N/S); and for the Lat-high and the Lat-low with the same range of altitudes (0−500 m a.s.l). The high- (low-) elevation stations were first grouped into 100-m-wide elevation bands starting at 500 m (0 m). The band anomaly values were then produced by simple averaging of individual station anomaly values within each band. Afterwards, the regional anomaly values were computed by re-averaging of the individual band anomaly values, and the regional trend was calculated as the slope of simple linear regression. A similar procedure was used for the comparison of temperature trends between the Lat-high and the Lat-low. The main difference is that the high- (low-) latitude stations were first grouped into 2-deg-wide latitude bands starting at 60°N (0°N).

### Analysis of elevation-dependent warming and latitude-dependent warming

This was performed for the Ele-high and the Ele-low as a whole, as well as for the Lat-high and the Lat-low as a whole, using a method similar to the elevation band method^15^,^16^ but with quantification of the amplification trend. For the high- and low- elevation (latitude) stations, the stations were first grouped into 100-m-wide altitudinal (2-deg-wide latitudinal) bands starting at 0 m (0 deg), the anomalies (relative to the 1961–2010 mean) for each band was then computed by simple averaging of individual station anomaly values, and the trend for that band was extracted from the average anomalies. Afterwards, the trend magnitude for each 500-m-wide altitudinal (10-deg-wide latitudinal) band was calculated by simple averaging of individual 100-m-wide altitudinal (2-deg-wide latitudinal) band trend magnitudes. The rate of altitudinal (latitudinal) amplification trend was computed as the gradient of the regression line for the trend magnitudes versus elevation (latitude).

### Analysis of relationships of *T*_t_ (*T*_b_) with altitude and latitude and of *T*_t_ with *T*_b_

The relationship of *T*_t_ (*T*_b_) with altitudes (*x*_1,_ in km) and latitudes (*x*_2,_ in km) was tested according to linear model of fit *T*_t_ (*T*_b_) = *b*_1_*x*_1_ + *b*_2_*x*_2_ + *c*, and nonlinear model of fit *T*_t_ (*T*_b_) = *b*_1_ *x*_1_ + *b*_2_ *x*_2_ + *b*_3_ (*x*_1_)^2^ + *b*_4_ (*x*_2_)^2^ + *b*_5_ (*x*_1_*x*_2_) + *c*, respectively. The relationship between *T*_t_ and *T*_b_ was tested according to model of fit *T*_t_ = *b*_0_*T*_b_ + *c*. Latitude in km = latitude in degree × *c*, where *c* is the distance constant (111.317 km degree^−1^) for each degree of latitude. The reason we have taken the regression coefficients (*b*_1_ and *b*_2_) in the linear model (*T*_t_ = *b*_1_*x*_1_ + *b*_2_*x*_2_ + *c*) as the rates of altitudinal and latitudinal amplification trends is that (1) the goodness of fit of the linear model is comparable to that of the non-linear model, judged from the multiple correlation coefficients (*R*); and (2) the statistical properties of the linear estimates are easier to determine relative to the non-linear estimates.

### Evaluation of the sensitivity of altitudinal amplification and latitudinal amplification trends to changes of altitude and number of available stations

This was carried out by repeatedly sampling the data of the Ele-high by omitting segments progressively from the lower limit of altitudinal gradient in 100 m steps, and computing the altitudinal amplification and latitudinal amplification trends as well as the related descriptive statistics for each altitude extent sampled (i.e., each subset of stations from the Ele-high). The altitudinal amplification and latitudinal amplification trends were estimated using the model of fit *T*_t_ = *b*_1_*x*_1_ + *b*_2_*x*_2_ + c as above. A similar method was used for the evaluation of the sensitivity of latitudinal amplification trend to changes of latitude and number of available stations (see Supplementary Table S4 for details on the difference).

To properly assess the effect of altitude (latitude), we proposed a new measure “warming signal quantity (WSQ)”. The WSQ for each altitude (latitude) extent was defined as the product of the average warming rate (°C) × the number of the stations for the altitude (latitude) extent. Meanwhile, three steps were taken to compute the altitudinal warming signal quantity (WSQ_alt_) and latitudinal warming signal quantity (WSQ_lat_) for each altitude extent where both altitudinal and latitudinal amplification trends can be detected. First the altitudinal warming component (*T*_talt_) and latitudinal warming component (*T*_tlat_) were computed as the products of the rate of altitudinal amplification trend × mean altitude, and the rate of latitudinal amplification trend × mean latitude, respectively. Second, the relative contributions of altitude and latitude (RC_ALT_ and RC_LAT_, respectively) were estimated using the formula RC_ALT_ = *T*_talt_/(*T*_talt_+ *T*_tlat_), and RC_LAT_ = *T*_tlat_/(*T*_talt_ + *T*_tlat_), respectively. Finally, the WSQ_alt_ and WSQ_lat_ were produced as the products of WSQ × RC_ALT_, and WSQ × RC_ALT_, respectively.

## Additional Information

**How to cite this article**: Wang, Q. *et al.* Evidence of high-elevation amplification versus Arctic amplification. *Sci. Rep.* **6**, 19219; doi: 10.1038/srep19219 (2016).

## Supplementary Material

Supplementary Information

## Figures and Tables

**Figure 1:**
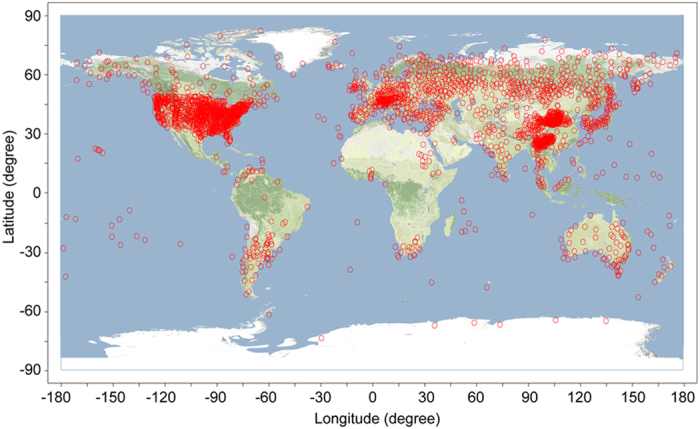
Distribution of 2781 stations used for this study around the globe. Of all the stations, 2598 (93.4%) are located in the Northern Hemisphere, and 183 (6.6%) are in the Southern Hemisphere. The map is generated with the Adobe Photoshop CS6 and the Golden Software Surfer 8.0.

**Table 1:** Linear models showing relationships of temperature trends with altitude and latitude, of temperature trends with base temperatures and of base temperatures with altitude and latitude for the four groups of stations tested.

Code	Station group	Model		*R/r*	*p*	n
		***T***_**t**_** = b**_**3**_ ***x***_**1**_** + b**_**4**_ ***x***_**2**_** + c**_**3**_				
A_1_	Ele-high	*T*_t_ = 0.2297 *x*_1_ + 0. 2510 × 10^−3^ *x*_2_ −0.0882	(1.1)	**0.434**	<0.0001	910
A_2_	Ele-low	*T*_t_ = 0. 1925 × 10^−3^ *x*_2_ + 0.3208	(2.1)	**0.443**	<0.0001	1732
C_1_	Arctic stations	*—*	(3.1)	***—***	*—*	187
C_2_	NH-Ele-low	*T*_t_ = 0. 1647 × 10^−3^ *x*_2_ + 0.4748	(4.1)	**0.425**	<0.0001	1715
		***T***_**t**_** = b**_**0**_***T***_**b**_** + c**_**1**_				
A_1_	Ele-high	*T*_t_ = −0.0488*T*_b_ + 1.6877	(1.2)	**−0.489**	<0.0001	910
A_2_	Ele-low	*T*_t_ = −0.0327 *T*_b_ + 1.5666	(2.2)	**−0.446**	<0.0001	1732
C_1_	Arctic stations	*T*_t_ = −0.0284 *T*_b_ + 1.4890	(3.2)	**−0.263**	=0.0003	187
C_2_	NH-Ele-low	*T*_t_ = −0.0286 *T*_b_ + 1.5474	(4.2)	**−0.440**	<0.0001	1715
		***T***_**b**_** = b**_**1**_ ***x***_**1**_** + b**_**2**_ ***x***_**2**_** + c**_**2**_				
A_1_	Ele-high	*T*_b_ = −4.2023 *x*_1_ −0.5510 × 10^−2^ *x*_2_ + 37.2173	(1.3)	**0.907**	<0.0001	910
A_2_	Ele-low	*T*_b_ = –0.5405 × 10^−2^ *x*_2_ + 35.9117	(2.3)	**0.912**	<0.0001	1732
C_1_	Arctic stations	*T*_b_ = −0.7110 × 10^−2^ *x*_2_ + 47.4941	(3.3)	**0.519**	<0.0001	187
C_2_	NH-Ele-low	*T*_b_ = −0.5458 × 10^−2^ *x*_2_ + 36.0333	(4.3)	**0.917**	<0.0001	1715

The models are established based on temperature trends (*T*_t_, in °C 50-yr^−1^), base temperatures (the base period 1961–1990 means, *T*_b_, in °C), altitudes (*x*_1_, in km), and latitudes (*x*_2_, in km) at individual stations using the stepwise regression or simple linear regression method according to models of fit *T*_*t*_ = *b*_3_*x*_1_ + *b*_4_*x*_2_ + *c*_3_, *T*_t_ = *b*_0_*T*_*b*_ + *c*_1_ and *T*_b_ = *b*_1_*x*_1_ + *b*_2_*x*_2_ + *c*_2_, respectively. Latitude in km = latitude in degree × *c*, where *c* is the distance constant (111.317 km degree^−1^) for each degree of latitude. Multiple/simple correlation coefficients (*R/r)* are given with two-tailed *p* values, and significant coefficients, at the 95% confidence level, are set in bold (see Supplementary Table S1 for details on the descriptive statistics of the four groups of stations).
