# Supplementary material for: Evidence of high-elevation amplification versus Arctic amplification
Source: Sci Rep. 2016 Jan 12;6:19219. doi: 10.1038/srep19219 (PMC4709741; doi:10.1038/srep19219)
Supplement: Supplementary Information [file srep19219-s1.pdf]

# Evidence of high-elevation amplification versus Arctic amplification

Qixiang Wang, Xiaohui Fan & Mengben Wang*

Institute of Loess Plateau, Shanxi University, Taiyuan 030006, China.

*Corresponding author: email: mbwang@sxu.edu.cn

## SUPPLEMENTARY INFORMATION

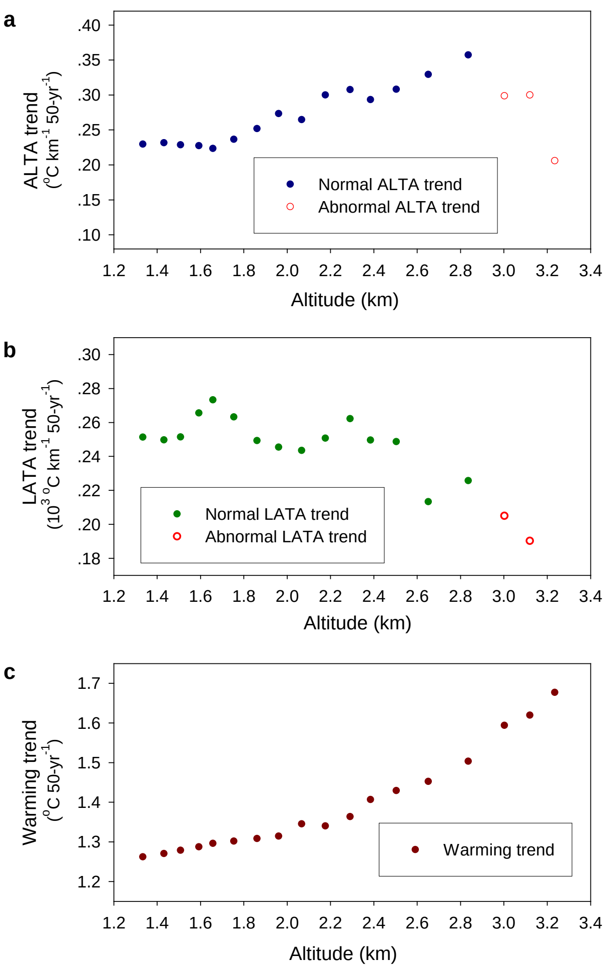


**Supplementary Figure S1** Changes of (**a**) altitudinal amplification (ALTA) trend, (**b**) latitudinal amplification (LATA) trend and (**c**) warming trend with altitude. The data are obtained from sampling the data of Ele-high by omitting segments from the lower limit of altitudinal gradient in 100 m steps (see **Supplementary Table S3** for details).

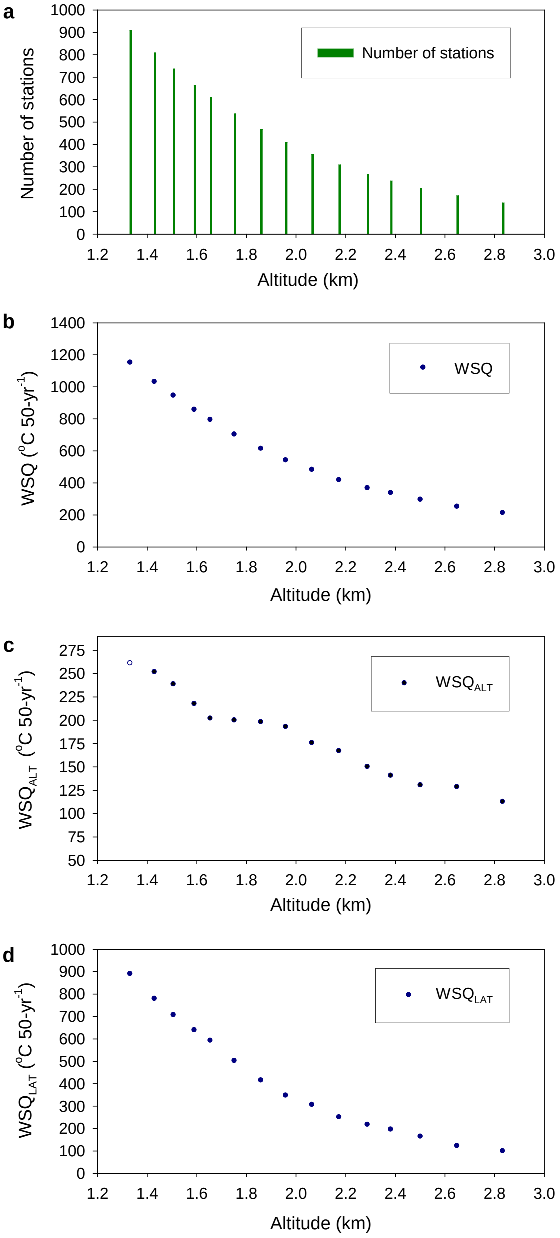


**Supplementary Figure S2** Changes of (**a**) number of stations, (**b**) total warming signal quantity (WSQ), (**c**) altitudinal warming signal quantity ($WSQ_{ALT}$), and (**d)** latitudinal warming signal quantity ($WSQ_{LAT}$) with altitude. The data on the number of stations are obtained from sampling the data of Ele-high by omitting segments from the lower limit of altitudinal gradient in 100 m steps (see **Supplementary Table S3** for details on the data, and **Methods** on calculations of WSQ, $WSQ_{ALT}$ and $WSQ_{LAT}$).

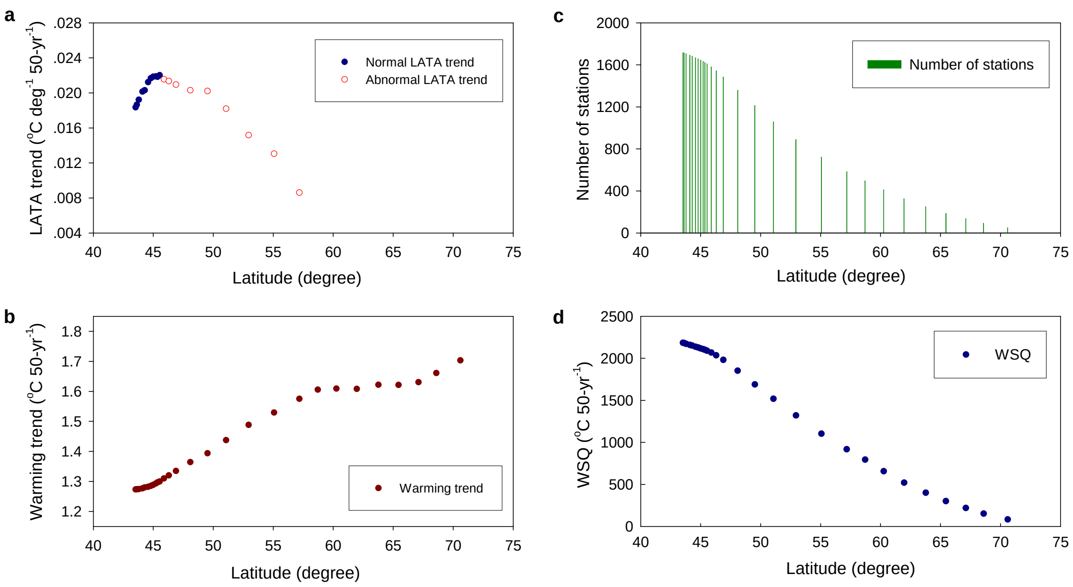


**Supplementary Figure S3** Changes of (**a**) latitudinal amplification (LATA) trend, (**b**) warming trend, (**c**) number of stations, and (**d**) the warming signal quantity (WSQ) with latitude. The data for plots **a-c** are obtained from sampling the data of NH-Ele-low by omitting segments from the lower limit of latitudinal gradient in 2.5-deg steps (see **Supplementary Table S4** for details on the data, and **Methods** on calculation of WSQ).

**Supplementary Table S1** Statistics of station altitude (in km) and latitude (in deg) for the six groups of stations used in this study.

| Scale | Code | Short name | Name of station group | Var. | Mean | Mini. | Max. | n |
|---|---|---|---|---|---|---|---|---|
| Global scale | $A_1$ | Ele-high | High-elevation stations (>500 m) across the globe | Alt. | 1.3334 | 0.5050 | 4.7000 | 910 |
| | | | | Lat. | 37.31 | 3.40 | 63.25 | |
| | $A_2$ | Ele-low | Low-elevation counterparts of Ele-high [low-elevation stations (≤500 m) located in the same latitudes] | Alt. | 0.1655 | 0.0010 | 0.5000 | 1732 |
| | | | | Lat. | 40.78 | 3.60 | 63.22 | |
| Northern Hemispheric scale | $B_1$ | Lat-high | Low-elevation stations (≤500 m) in the Arctic region | Alt. | 0.0927 | 0.0020 | 0.3990 | 184 |
| | | | | Lat. | 65.48 | 60.13 | 82.05 | |
| | $B_2$ | Lat-low | Lower latitude counterparts of Lat-high [lower latitude stations (≤60 °N) in the same altitudes] | Alt. | 0.1734 | 0.0010 | 0.5000 | 1531 |
| | | | | Lat. | 40.89 | 1.48 | 60.00 | |
| | $C_1$ | Arctic stations | Stations in the Arctic region (North of 60 °N) | Alt. | 0.1020 | 0.0020 | 0.7410 | 187 |
| | | | | Lat. | 65.43 | 60.05 | 82.05 | |
| | $C_2$ | NH-Ele-low | Low-elevation stations (≤500 m) across the Northern Hemisphere | Alt. | 0.1648 | 0.0010 | 0.5000 | 1715 |
| | | | | Lat. | 43.53 | 1.48 | 82.05 | |
| Global scale | | All stations | All available stations around the globe | Alt. | 0.5429 | 0.0010 | 4.7000 | 2781 |
| | | | | Lat. | 40.81 | 1.48 | 82.50 | |

**Supplementary Table S2** Number of stations with significant positive (SP), non-significant positive (NSP), positive (SP+NSP), significant negative (SN), non-significant negative (NSN), and negative (SN+NSN) trends for the six groups of stations used in this study.

| Scale | Code | Short name | SP | NSP | Positive | SN | NSN | Negative | n |
|---|---|---|---|---|---|---|---|---|---|
| Global scale | A1 | Ele-high | 786 (86.4) | 118 (13.0) | 904 (99.4) | 3 (0.3) | 3 (0.3) | 6 (0.6) | 910 |
| | A2 | Ele-low | 1462 (84.4) | 239 (13.8) | 1701 (98.2) | 3 (0.2) | 28 (1.6) | 31 (1.8) | 1732 |
| Northern | B1 | Lat-high | 137 (74.5) | 44 (23.9) | 181 (98.4) | | 3 (1.6) | 3 (1.6) | 184 |
| Hemispheric | B2 | Lat-low | 1328 (86.7) | 182 (11.9) | 1510 (98.6) | 2 (0.1) | 19 (1.2) | 21 (1.4) | 1531 |
| scale | C1 | Arctic stations | 139 (74.3) | 45 (24.1) | 184 (98.4) | | 3 (1.6) | 3 (1.6) | 187 |
| | C2 | NH-Ele-low | 1465 (85.4) | 226 (13.2) | 1691 (98.6) | 2 (0.1) | 22 (1.3) | 24 (1.4) | 1715 |
| Global scale | | All stations | 2348 (84.4) | 395 (14.2) | 2743 (98.6) | 6 (0.2) | 32 (1.2) | 38 (1.4) | 2781 |

The trend for each station was extracted from the anomalies (relative to the 1961-1990 mean) using the least squares best-fit method. The trend significance is judged using 95% confidence level. The value in parentheses represents the percentage of the number of the stations relative to the total number of the stations of each group.

**Supplementary Table S3** Changes of altitudinal amplification trend and latitudinal amplification trend derived from sampling the data of Ele-high by omitting segments from the lower limit of altitudinal gradient in 100 m steps.

| **Sample** | **Alt. extent** | **Model ($T_t = a\,X_1 + b\,X_2 + c$)** | | | | | **Average Alt.** | **Average $T_t$** | **No. of stations** | **WSQ** |
|---|---|---|---|---|---|---|---|---|---|---|
| | | ***a*** | ***b*×10^3** | ***c*** | ***R*** | ***p*** | | | | |
| Entire | 0.5-4.7 km | 0.2297 | 0.2513 | -0.0882 | **0.434** | <0.0001 | 1.3334 | 1.262 | 910 | 1148.4 |
| 1 | 0.6-4.7 km | 0.2315 | 0.2497 | -0.0860 | **0.430** | <0.0001 | 1.4313 | 1.271 | 809 | 1028.2 |
| 2 | 0.7-4.7 km | 0.2287 | 0.2515 | -0.0861 | **0.424** | <0.0001 | 1.5078 | 1.279 | 737 | 942.6 |
| 3 | 0.8-4.7 km | 0.2275 | 0.2656 | -0.1382 | **0.440** | <0.0001 | 1.5922 | 1.287 | 663 | 853.3 |
| 4 | 0.9–4.7 km | 0.2235 | 0.2733 | -0.1581 | **0.448** | <0.0001 | 1.6568 | 1.296 | 610 | 790.6 |
| 5 | 1.0–4.7 km | 0.2365 | 0.2632 | -0.1521 | **0.441** | <0.0001 | 1.7532 | 1.302 | 537 | 699.2 |
| 6 | 1.1–4.7 km | 0.2519 | 0.2493 | -0.1390 | **0.463** | <0.0001 | 1.8605 | 1.308 | 466 | 609.5 |
| 7 | 1.2–4.7 km | 0.2732 | 0.2454 | -0.1822 | **0.483** | <0.0001 | 1.9606 | 1.314 | 409 | 537.4 |
| 8 | 1.3–4.7 km | 0.2647 | 0.2435 | -0.1503 | **0.472** | <0.0001 | 2.0664 | 1.345 | 356 | 478.8 |
| 9 | 1.4–4.7 km | 0.2999 | 0.2507 | -0.2833 | **0.509** | <0.0001 | 2.1756 | 1.340 | 309 | 414.1 |
| 10 | 1.5–4.7 km | 0.3077 | 0.2622 | -0.3510 | **0.522** | <0.0001 | 2.2899 | 1.363 | 267 | 363.9 |
| 11 | 1.6–4.7 km | 0.2934 | 0.2496 | -0.2575 | **0.489** | <0.0001 | 2.3841 | 1.407 | 237 | 333.5 |
| 12 | 1.7–4.7 km | 0.3080 | 0.2487 | -0.3024 | **0.485** | <0.0001 | 2.5032 | 1.429 | 204 | 291.5 |
| 13 | 1.8–4.7 km | 0.3293 | 0.2133 | -0.2417 | **0.471** | <0.0001 | 2.6512 | 1.453 | 171 | 248.5 |
| 14 | 1.9–4.7 km | 0.3571 | 0.2257 | -0.3856 | **0.470** | <0.0001 | 2.8351 | 1.503 | 139 | 208.9 |
| 15 | 2.0–4.7 km | 0.2986 | 0.2050 | -0.0976 | **0.381** | =0.0001 | 3.0019 | 1.594 | 117 | 186.5 |
| 16 | 2.1–4.7 km | 0.2999 | 0.1903 | -0.0470 | **0.363** | =0.0008 | 3.1197 | 1.620 | 104 | 168.5 |
| 17 | 2.2–4.7 km | 0.2060 | – | 1.0108 | **0.226** | =0.0293 | 3.2343 | 1.677 | 93 | 156.0 |
| 18 | 2.3–4.7 km | – | – | – | – | – | 3.3142 | 1.718 | 86 | 147.7 |

The model between temperature trends ($T_t$, in °C 50-yr$^{-1}$) and altitudes ($x_1$, in km) and latitudes ($x_2$, in km) for each sample is determined using the stepwise regression method. Multiple correlation coefficient ($R$) is given with two-tailed $p$ value in next column. Significant coefficient, at the 95 % confidence level, is set in bold. For samples 15 to 17, the multiple correlation coefficients (as well as the rates of altitudinal amplification trend and latitudinal amplification trend) are obviously smaller than those just before; and for sample 18, neither altitudinal amplification trend nor latitudinal amplification trend can be detected further. Also shown are the average altitude (km), average $T_t$ (°C 50-yr$^{-1}$), number of stations, and warming signal quantity (°C 50-yr$^{-1}$, WSQ) for each sample.

**Supplementary Table S4** Change of latitudinal amplification trend derived from sampling the data of NH-Ele-low by omitting segments from the lower limit of latitudinal gradient in 2.5-deg steps.

| **Sample** | **Latitude extent** | **Model ($T_t = b\,x_2 + c$)** | | | | **Average lat.** | **Average $T_t$** | **No. of stations** | **WSQ** |
|---|---|---|---|---|---|---|---|---|---|
| | | $b \times 10^3$ | $c$ | $r$ | $p$ | | | | |
| Entire | 0°–82.5°N | 0.1647 | 0.4748 | **0.425** | <0.0001 | 43.53 | 1.273 | 1715 | 2183.1 |
| 1 | 2.5°–82.5° N | 0.1675 | 0.4602 | **0.428** | <0.0001 | 43.62 | 1.274 | 1711 | 2179.0 |
| 2 | 5.0°– 82.5° N | 0.1726 | 0.4330 | **0.433** | <0.0001 | 43.79 | 1.274 | 1704 | 2171.5 |
| 3 | 7.5°–82.5° N | 0.1809 | 0.3885 | **0.438** | <0.0001 | 44.10 | 1.277 | 1690 | 2157.6 |
| 4 | 10.0°–82.5° N | 0.1823 | 0.3814 | **0.433** | <0.0001 | 44.29 | 1.280 | 1681 | 2151.5 |
| 5 | 12.5°– 82.5° N | 0.1906 | 0.3363 | **0.439** | <0.0001 | 44.57 | 1.282 | 1667 | 2137.0 |
| 6 | 15.0°–82.5° N | 0.1947 | 0.3139 | **0.438** | <0.0001 | 44.79 | 1.285 | 1655 | 2126.3 |
| 7 | 17.5°–82.5° N | 0.1963 | 0.3055 | **0.434** | <0.0001 | 45.00 | 1.289 | 1643 | 2117.2 |
| 8 | 20.0°–82.5° N | 0.1966 | 0.3037 | **0.430** | <0.0001 | 45.21 | 1.293 | 1630 | 2107.7 |
| 9 | 22.5°–82.5° N | 0.1961 | 0.3063 | **0.424** | <0.0001 | 45.36 | 1.297 | 1620 | 2100.5 |
| 10 | 25.0°–82.5° N | 0.1979 | 0.2966 | **0.422** | <0.0001 | 45.53 | 1.299 | 1607 | 2088.2 |
| 11 | 27.5°–82.5° N | 0.1935 | 0.3213 | **0.406** | <0.0001 | 45.88 | 1.310 | 1578 | 2066.8 |
| 12 | 30.0°–82.5° N | 0.1917 | 0.3317 | **0.395** | <0.0001 | 46.30 | 1.320 | 1541 | 2033.9 |
| 13 | 32.5°–82.5° N | 0.1882 | 0.3527 | **0.379** | <0.0001 | 46.89 | 1.335 | 1483 | 1979.5 |
| 14 | 35.0°–82.5° N | 0.1823 | 0.3882 | **0.349** | <0.0001 | 48.09 | 1.364 | 1357 | 1851.2 |
| 15 | 37.5°–82.5° N | 0.1815 | 0.3934 | **0.330** | <0.0001 | 49.51 | 1.394 | 1211 | 1687.9 |
| 16 | 40.0°–82.5° N | 0.1634 | 0.5086 | **0.285** | <0.0001 | 51.07 | 1.437 | 1056 | 1517.8 |
| 17 | 42.5°–82.5° N | 0.1364 | 0.6845 | **0.226** | <0.0001 | 52.94 | 1.488 | 887 | 1319.9 |
| 18 | 45.0°–82.5° N | 0.1173 | 0.8105 | **0.178** | <0.0001 | 55.07 | 1.529 | 721 | 1102.7 |
| 19 | 47.5°–82.5° N | 0.0773 | 1.0825 | **0.109** | =0.0084 | 57.18 | 1.575 | 582 | 916.5 |
| 20 | 50.0°–82.5° N | - | - | - | - | 58.71 | 1.605 | 494 | 793.1 |

The model between temperature trends ($T_t$, in °C 50-yr$^{-1}$) and latitudes ($x_2$, in km) is determined using the simple linear regression method. Pearson correlation coefficient ($r$) is given with two-tailed $p$ value in next column. Significant coefficient, at the 95 % confidence level, is set in bold. For samples 11 to 19, the correlation coefficients (as well as the rates of latitudinal amplification trends) are obviously smaller than those just before; and for sample 20, no latitudinal amplification trend can be detected further. Also shown are the average latitude (deg), average $T_t$ (°C 50-yr$^{-1}$), number of stations, and warming signal quantity (°C 50-yr$^{-1}$, WSQ) for each sample.
